# Surgical Extraction of Traumatic Lodgment of a Pen Nib Between the Eye and Nasal Bridge of an Infant: A Case Report

**DOI:** 10.7759/cureus.28477

**Published:** 2022-08-27

**Authors:** Krishna Shah, Vaishali Prajapati, Smeet Desai, Karishma Dhaval Desai, Mayur B Wanjari, Deepika Singhal

**Affiliations:** 1 Ophthalmology, GMERS Medical College and Civil Hospital, Sola, Ahmedabad, IND; 2 Ophthalmology, Surat Municipal Institute of Medical Education and Research (SMIMER), Surat, IND; 3 Research, Jawaharlal Nehru Medical College, Datta Meghe Institute of Medical Sciences (Deemed to be University), Wardha, IND

**Keywords:** emergency surgery, eye injuries, surgical extraction, nib of pen, child trauma

## Abstract

Unintentional eye injuries are common in small children. If left unobserved by parents or caretakers, it is challenging to assess as the kids are in distress and pain. Penetrating trauma in or near the eye requires urgent treatment as it can lead to infection and other complications. Early diagnosis and management help avoid further complications. An 11-month-old female was presented by her parents at a tertiary care clinic with inconsolable crying and swelling over the right eye's upper lid for one day. There was a history of possible trauma with a pen while the child was playing. During the examination, swelling in the right periorbital region between the eye and nasal bridge with the opening of the foreign body tract was noted. A skull X-ray with orbit showed a radiopaque nib of the pen in the right periorbital soft tissue. Emergency surgery was planned under general anesthesia. Surgery was performed and the pen was extracted from the right periorbital soft tissue lying between the eye and nasal bridge. Parents and health care providers, including pediatricians, should assess a crying child with a trauma history carefully even with no apparent clinical findings. Efforts must be done to correlate clinical findings with proper history and other needed investigations.

## Introduction

Any eye injury or adnexa injury in an infant is an emergency, and prompt diagnosis and treatment are required [1]. The diagnosis is difficult as the infant is distressed and unable to communicate [1-2]. Evidence suggests that the intervention must manage such a foreign body [3]. This requires urgency and any penetrating foreign body in or near the eye requires urgent removal of foreign material if possible to avoid further complications [4-6].

## Case presentation

An 11-month-old female was presented by her parents at the nearest primary health center (PHC) center with a complaint of inconsolable crying and swelling near the right upper eye lid. At the primary care unit, the child received a prescription of eye drops and analgesic medication. The patient was referred to an advanced medical setting for further management. The patient went to private providers, including private hospitals and the diagnosis was a suspected penetrating injury near the eye. With no conclusive diagnosis, the patient was referred to a tertiary care teaching hospital a day after the history of trauma. There was a history of trauma near the right eye before 24 hours. Before visiting the tertiary care center, the baby was examined by various health care providers, including ENT, a general practitioner, and a pediatrician; however, no diagnostic or radiological investigation was prescribed. 

The child was in severe distress, irritable and inconsolable crying during the examination of the patient. On inspection, between the right eye and nasal bridge foreign body tract opening was noted with edema and ecchymosis surrounding it. There was no significant systemic illness.

**Figure 1:**
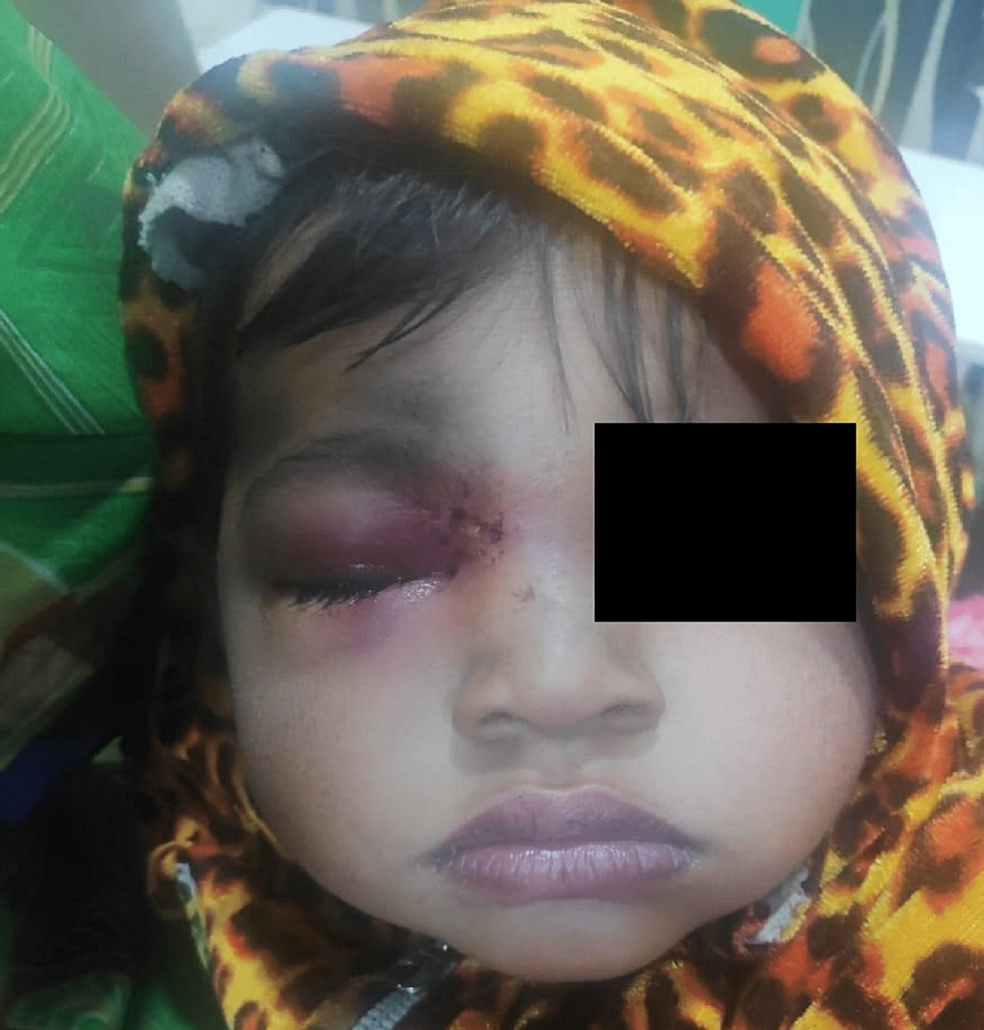
Showing Opening of Foreign Body Tract Between Eye and Nasal Bridge.

**Figure 2:**
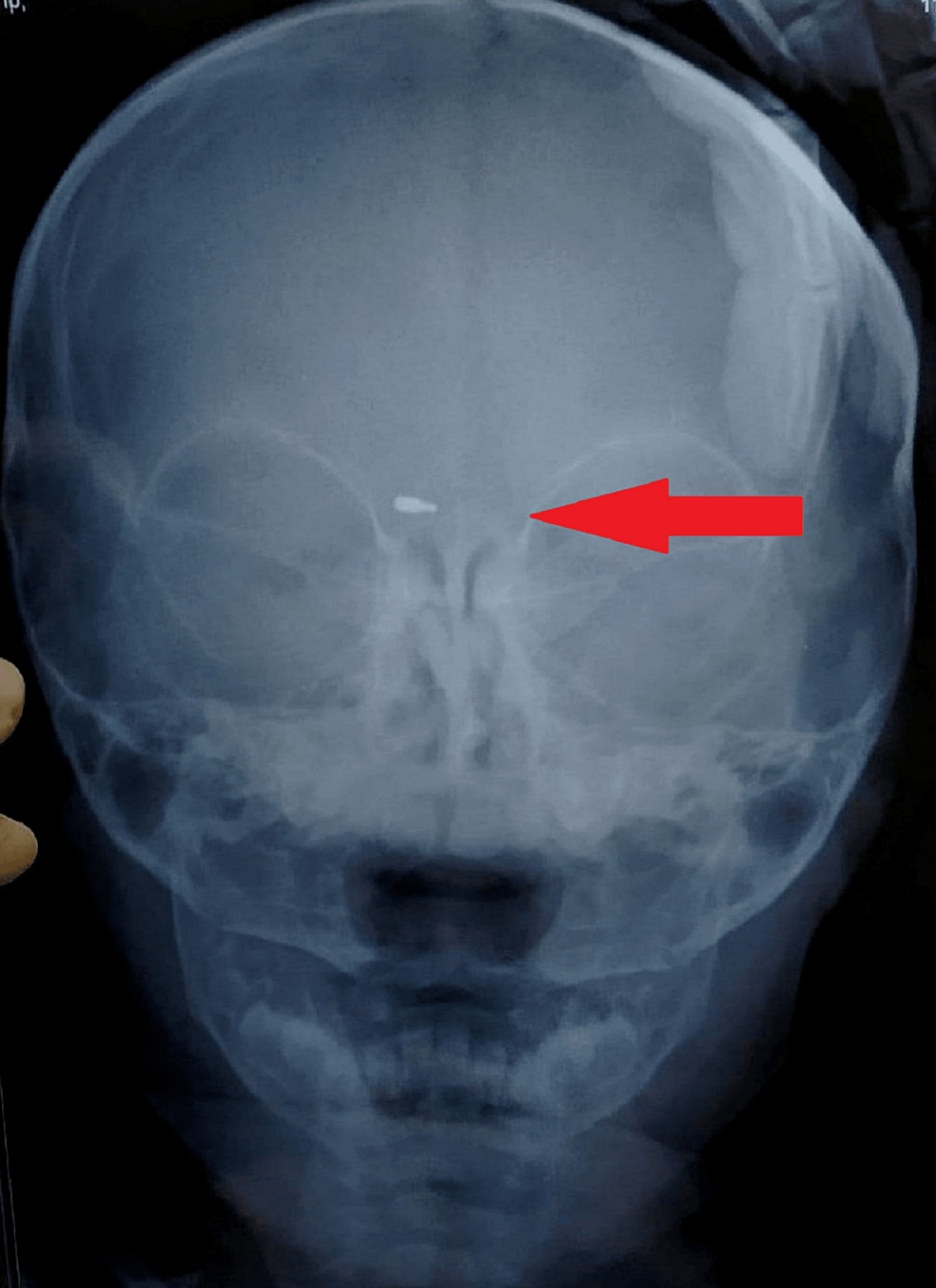
Showing Radiopaque Foreign Material in Right Periorbital Soft Tissue.

The baby was sent to the ophthalmology department for diagnostic assessment in the tertiary care clinic. A clinical ophthalmic examination revealed an inflamed eye (Figure 1), suggesting pre-septal cellulitis. Routine blood investigations along with an X-ray skull with orbit were advised with a possibility of doubt of lodgment of foreign material. On X-ray, the nib of a pen was found as radiopaque material in the right periorbital soft tissue around 2.5 cm deep from the surface (Figure 2). It was decided to undertake surgical removal of the nib as the surgery was to be performed under general anesthesia, so pediatric and anesthetic fitness was taken along with other routine pre-anesthetic investigations.

Pre-operative intravenous injectable antibiotics were given according to the weight of the child. Surgery was performed to remove the pen nib and the exact site location and the depth of lodgment of the nib of the pen were accessed with the help of a plain X-ray. The incision was made and dissection was done until the pen nib was found. The nib was extracted with the help of artery forceps. Sutures were taken to close the wound. 

For the follow-up and outcome, the patient was kept under examination for two days after the surgery. Post-discharge, the patient was advised to follow up after one week. Compliance with advice was satisfactory by the patient’s parents. Post-discharge compliance was checked through telephone calls. 

## Discussion

The incidence of intraorbital foreign bodies during orbital trauma is 2.9%, and ocular trauma is a significant contributor to ocular morbidity in children. Falls are the most common cause of juvenile ocular trauma, accounting for 37% of all cases [7]. Long after the accident, an unidentified foreign body can cause issues. CT is the preferred imaging technique for detecting intraorbital foreign entities. Magnetic resonance imaging (MRI) is not always available in an emergency and is contraindicated when a metallic intraorbital foreign body is suspected [8,9].

The nature and position of the foreign body in the orbit influence surgical removal. Because of the increased risk of inflammation and infection, it is generally suggested for all organic foreign bodies. It is also used to treat intraorbital foreign bodies that obstruct globe motion or are aggravated by infection, as well as exophthalmos or optic nerve compression [10].

Foreign bodies lying posterior to the orbit are more likely to cause oculomotor dysfunction and visual neuropathy following surgical removal, whereas anteriorly placed foreign bodies are easier to remove, and removal of an anteriorly located metallic foreign body enables MRI [8]. Extraction is also appropriate when there is a large, sharp foreign body present, which was the surgical indication in our observation, especially when there was uncertainty regarding the foreign body's precise nature [9].

Early diagnosis and management are important in childhood trauma as it is often neglected because of parents’ lack of awareness. Once the diagnosis is confirmed, no delay in treatment is a goal to achieve a better outcome [5]. Penetrated foreign material can infect the surrounding area and involve deeper structures. Extracting foreign material as early as possible can avoid such complications [6].

## Conclusions

This present case describes accidental traumatic lodgment of the metallic tip of a ball pen near the eye just above the lachrymal area. As the baby was profusely crying and rubbing the eye, the injury site was left unnoticed and led to misdiagnosis with an eye injury. The patient was clinically examined by various health care providers, including ophthalmologists, ENT surgeons and a pediatrician. But the nib of the pen lodged between the bridge of the eye and nose was left unnoticed by the examining medical professionals or health providers. This is not unusual and is often overlooked. However, an anteroposterior and lateral x-ray of the face revealed a well-defined radiopaque foreign material lodged in the periorbital soft tissue between the eye and nose. Unintentional eye injuries in small children are prevalent. If left unobserved by parents or caretakers, it is challenging to assess as the kids are in distress and pain. Penetrating trauma in or near the eye requires urgent treatment as it can lead to infection and other complications. Early diagnosis and management are helpful to avoid further complications. Parents health care providers and pediatricians should give thorough attention to a crying child with a history of trauma with no apparent clinical findings. Efforts must be made to correlate clinical findings with proper history and other needed investigations. Avoid delaying the management to reduce complications. 
